# Adaptive Filtering Improved Apnea Detection Performance Using Tracheal Sounds in Noisy Environment: A Simulation Study

**DOI:** 10.1155/2020/7429345

**Published:** 2020-05-21

**Authors:** Yanan Wu, Jing Liu, Baolin He, Xiaotong Zhang, Lu Yu

**Affiliations:** ^1^Department of Biomedical Engineering, School of Fundamental Sciences, China Medical University, Shenyang, Liaoning, China; ^2^Department of Nuclear Medicine, Zhongnan Hospital of Wuhan University, Wuhan, Hubei, China

## Abstract

**Objective:**

Tracheal sounds were used to detect apnea on various occasions. However, ambient noises can contaminate tracheal sounds which result in poor performance of apnea detection. The objective of this paper was to apply the adaptive filtering (AF) algorithm to improve the quality of tracheal sounds and examine the accuracy of the apnea detection algorithm using tracheal sounds after AF.

**Method:**

Tracheal sounds were acquired using a primary microphone encased in a plastic bell, and the ambient noises were collected using a reference microphone resting outside the plastic bell in quiet and noisy environments, respectively. Simultaneously, the flow pressure signals and thoracic and abdominal movement were obtained as the standard signals to determine apnea events. Then, the normalized least mean square (NLMS) AF algorithm was applied to the tracheal sounds mixed with noises. Finally, the algorithm of apnea detection was used to the tracheal sounds with AF and the tracheal sounds without AF. Sensitivity, specificity, positive predictive value (PPV), negative predictive value (NPV), accuracy, and Cohen's kappa coefficient of apnea detection were calculated.

**Results:**

Forty-six healthy subjects, aged 18-35 years and with BMI < 21.4, were included in the study. The apnea detection performance using tracheal sounds was as follows: in the quiet environment, the tracheal sounds without AF detected apnea with 97.2% sensitivity, 99.9% specificity, 99.8% PPV, 99.4% NPV, 99.5% accuracy, and 0.982 kappa coefficient. The tracheal sounds with AF detected apnea with 98.2% sensitivity, 99.9% specificity, 99.4% PPV, 99.6% NPV, 99.6% accuracy, and 0.985 kappa coefficient. While in the noisy environment, the tracheal sounds without AF detected apnea with 81.1% sensitivity, 96.9% specificity, 85.1% PPV, 96% NPV, 94.2% accuracy, and 0.795 kappa coefficient and the tracheal sounds with AF detected apnea with 91.5% sensitivity, 97.4% specificity, 88.4% PPV, 98.2% NPV, 96.4% accuracy, and 0.877 kappa coefficient.

**Conclusion:**

The performance of apnea detection using tracheal sounds with the NLMS AF algorithm in the noisy environment proved to be accurate and reliable. The AF technology could be applied to the respiratory monitoring using tracheal sounds.

## 1. Introduction

Tracheal sounds have received much attention in recent years and are often used on respiration-related occasions, such as detecting apnea in sedated volunteers [1] and postanesthesia care unit patients [2], screening obstructive sleep apnea (OSA) during wakefulness [3–6] and detecting OSA during sleep [7–10]. OSA, which increases risk of cardiocerebral vascular disease [11], depression [12], diabetes [13], and even traffic accidents [14], is characterized by repetitive upper airway obstruction occurring during sleep [15]. However, the foremost problem is that talking, machine alarms in the postanesthesia care unit, and ambient noises during wakefulness or sleep are still interferences when tracheal sounds are acquired. A quiet environment on these occasions is frequently highly difficult to maintain. Hence, a simple and effective denoising algorithm is needed.

Adaptive filtering (AF), which can deal with various kinds of signals in unknown statistical environment or in nonstationary environment, is a promising method of signal processing in adaptive noise cancellation [16]. It is usually better than a fixed filter designed through conventional methods and has been widely used [17], such as removing artifacts in electroencephalography (EEG) [18, 19], electrocardiography (ECG) [20], impedance cardiography [21], and photoplethysmography (PPG) [22]. However, the accuracy and reliability of the apnea detection algorithm using tracheal sounds after AF have not been examined. In this study, our hypothesis was that AF could effectively improve the quality of recorded tracheal sounds and result in better performance of apnea detection.

## 2. Materials and Methods

### 2.1. Experiment Procedure and Data Collection

Forty-six healthy male (*n* = 24) and female volunteers (*n* = 22), aged 18-35 yr and with BMI < 21.4, were enrolled in the study after approval for the study protocol was obtained from the Human Institutional Review Board of the China Medical University. All subjects gave informed consent before the measurements. The study was registered at Chinese Clinical Trial Registry (http://www.chictr.org.cn/) under number ChiCTR-DDD-17014238.

The acquisition procedure of tracheal sounds and nasal flow pressure signals was as follows: The subjects in the supine position were asked to breathe normally under resting states for 2 minutes. Then, the subjects were asked to hold their breath for about 20 sec to simulate an event of apnea. This type of apnea simulation was repeated every 40 sec for 10 times. For every subject, the total duration was about 15 minutes. The procedure above was performed in a quiet environment and noisy environment where a speaker randomly played some TV dramas, respectively. Our pilot study determined the volume of the speaker, which produced missed events of apnea detected by tracheal sounds. The tracheal sounds and polysomnography (PSG) signals from the subjects were recorded simultaneously during the whole experiment.

**Figure 1:**
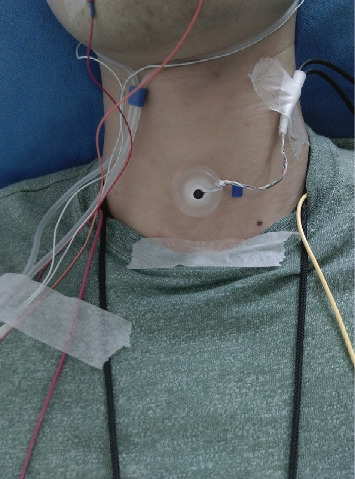
The plastic bell over the trachea.

**Figure 2:**
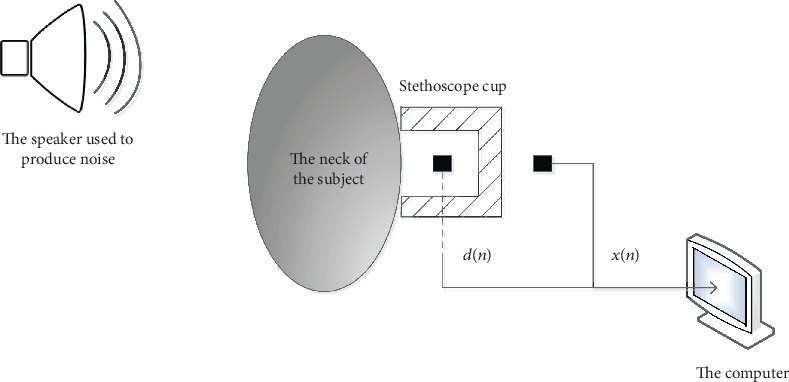
The setup of tracheal sound acquisition.

The tracheal sounds from the subjects were acquired in the supine position, using a microphone (HC4015G-02L25-423, Hong Chang Electronics, Shenzhen, China) encased in a plastic bell. The bell was attached to the subject's neck by a double-stick disc just below the larynx and above the suprasternal notch, as is shown in Figure 1. The primary microphone encased in the bell provided the tracheal sounds mixed with noise as the primary input for AF. The secondary microphone resting outside the bell provided the ambient noise as the reference input for AF. A diagram of this setup is illustrated in Figure 2. The audio signals were continuously recorded on the computer using the software Adobe Audition CC 2018 (Adobe Inc., San Jose, California, USA) at 22050 Hz.

Each subject was fitted with a series of sensors connected to a PSG (Alice PDx, Amsterdam, Holland) to measure nasal flow pressure, thoracic movement, abdominal movement, etc. PSG is the gold standard for diagnosing sleep apnea but has not been widely used because of the high cost. In the experiment, flow pressure signals and thoracic and abdominal movement were used as the gold standard signals (PSG deriving signals) to determine apnea events. The recorded PSG signals were transferred to the computer. The acoustic and PSG data on the computer were processed and analyzed in Matlab R2017a (MathWorks Inc., Natick, MA). In our study, the absence of nasal flow pressure and thoracic and abdominal movement signals for more than 15 sec was considered an event of apnea.

### 2.2. The Implementation of Adaptive Filtering

**Figure 3:**
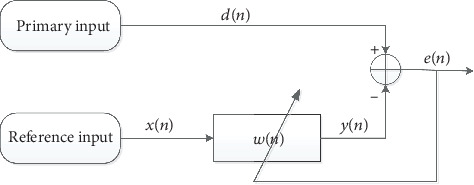
Block diagram of adaptive filter.

AF is a signal processing method that uses multiple signal sources to produce desired signals. The block diagram of AF is shown in Figure 3. As is shown in Figure 3, there are two inputs: the primary input *d*(*n*) and the reference input *x*(*n*). The signal *x*(*n*) is filtered by the FIR filter whose coefficient is *w*(*n*) to generate the signal *y*(*n*). The error signal, *e*(*n*), is calculated by the primary signal *d*(*n*) subtracting the filtered signal *y*(*n*). Then, the error signal *e*(*n*) and the reference input signal *x*(*n*) are used to update the coefficient *w*(*n*) using the normalized least mean square (NLMS) algorithm which changes its step size according to the reference input signal. Hence, the NLMS AF algorithm is suitable for a nonstationary environment and provides a trade-off in convergence and computational complexity. The equations used in this algorithm are

(1) $$\begin{array}{c} y\left(n\right)=x^{T}\left(n\right)*w\left(n\right), \end{array}$$

(2) $$\begin{array}{c} e\left(n\right)=d\left(n\right)-y\left(n\right), \end{array}$$

(3) $$\begin{array}{c} w\left(n+1\right)=w\left(n\right)+\frac{\mu }{x^{T}\left(n\right)x\left(n\right)+\psi }e\left(n\right)x\left(n\right), \end{array}$$

where *μ* is the step size parameter and *ψ* is a small value in order to ensure that the denominator of the equation is never zero.

In our study, signal *d*(*n*) (primary input) was the tracheal sounds mixed with noise and signal *x*(*n*) (reference input) was the ambient noise. Signal *e*(*n*) was the desired signals, the filtered tracheal sounds. The parameters in the NLMS AF algorithm were optimized in our pilot study. The value of *μ* was 0.08, *ψ* was 0.02, and the order of the filter was 64, respectively.

### 2.3. Evaluation Metrics of the Performance of Adaptive Filtering Algorithm

#### 2.3.1. The Synthetic Signal Simulation and Signal-to-Noise Ratio (SNR) Value Calculation

In this section, a simulation experiment was performed on the synthetic acoustic signal using the NLMS AF algorithm. In the simulation, we mixed the two signals: the 20 sec ambient noise signal *x*(*n*), which was chosen randomly from the secondary reference microphone in the simulated noisy environment, and the 20 sec uncontaminated tracheal sound *B*(*n*), which was chosen randomly from the primary microphone in the quiet environment. The two signals were synthesized into the mixed signal *s*(*n*) in the following way:

(4) $$\begin{array}{c} s\left(n\right)=B\left(n\right)+\text{Gi}*x\left(n\right), \end{array}$$

where Gi defined the proportion of noise in *s*(*n*) and was set to 1, 1.5, and 2, respectively. The NLMS AF algorithm was applied to *s*(*n*) with Gi∗*x*(*n*). In order to investigate the performance of the proposed filtering algorithm, we calculated the SNRo (before filtering) and the SNRf (after filtering) of the synthetic signal *s*(*n*) (Gi = 1, 1.5, 2). SNRo was defined as

(5) $$\begin{array}{c} \text{SNRo}=10*\log_{10}\left(\frac{\mathrm{var}\left(B\left(n\right)\right)}{\mathrm{var}\left(s\left(n\right)-B\left(n\right)\right)}\right), \end{array}$$

where var represented the variance transformation. SNRf was defined as

(6) $$\begin{array}{c} \text{SNRf}=10*\log_{10}\left(\frac{\mathrm{var}\left(B\left(n\right)\right)}{\mathrm{var}\left(e\left(n\right)-B\left(n\right)\right)}\right), \end{array}$$

where *e*(*n*) was the filtered tracheal sounds (error signal in AF system) and var represented the variance transformation.

#### 2.3.2. The Performance Presentation of Apnea Detection Using Tracheal Sounds

**Figure 4:**
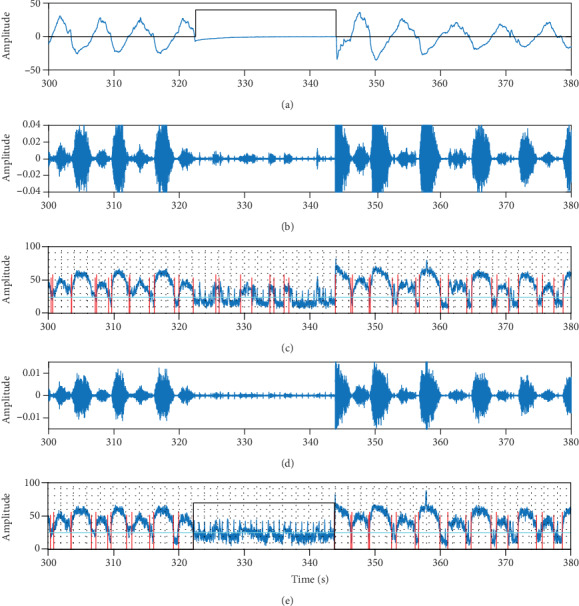
(a) Nasal flow pressure signal; the black rectangle represents apnea detected. (b) Tracheal sounds without AF in the noisy environment. (c) log-var signal without AF in the noisy environment; the light blue line represents the calculated log-var threshold, the red lines mark the beginning and end of inspiration or expiration, and the black rectangle represents apnea detected. (d) Tracheal sounds with AF in the noisy environment. (e) log-var signal with AF in the noisy environment; the light blue line represents the calculated log-var threshold, the red lines mark the beginning and end of inspiration or expiration, and the black rectangle represents apnea detected.

The detection of apnea was performed using two kinds of tracheal sounds, each in two different ambient noise conditions: the tracheal sounds before and after being processed by the AF algorithm in the quiet environment and the tracheal sounds before and after being processed by AF algorithm in the noisy environment. The apnea detection algorithm has been described in our previous study [2]. The tracheal sounds were initially filtered using a 5th-order Butterworth filter with a passband between 150 and 800 Hz to minimize heart sounds, muscle interference, and high-frequency noise. The processed signal was segmented into windows of 20 ms with 75% overlap between adjacent windows. The logarithm of the tracheal sound variance (log-var) in each window was calculated. Then, using a calculated log-var threshold, an inspiration or expiration was marked when the log-var signal crossed the threshold and lasted for at least 0.5 sec [2]. The absence of inspiration or expiration for more than 15 sec was considered an event of apnea.  Figure 4 showed the identified apneic periods based on PSG deriving signal (nasal flow pressure signal) and tracheal sounds.

Apnea detection performance using tracheal sounds was assessed by comparing it with apnea events detected by the PSG deriving signals. A true positive (TP) was defined if apnea was detected from the tracheal sounds and the PSG deriving signals. A false negative (FN) was defined if apnea was detected from the PSG deriving signals but not from tracheal sounds. False positive (FP) meant that apnea was detected from the tracheal sounds but not from the PSG deriving signals, and true negative (TN) meant that apnea was detected from neither PSG deriving signals nor tracheal sounds. The amount of time in which neither the acoustic apnea detection method nor the gold standard apnea detection method detected apnea was divided by 15 sec to calculate a value for the number of TN. Sensitivity (TP/[TP + FN]), specificity (TN/[TN + FP]), positive predictive value (PPV = TP/[TP + FP]), negative predictive value (NPV = TN/[TN + FN]), and accuracy ([TN + TP]/[TN + TP + FN + FP]) were calculated. Cohen's kappa coefficient was also calculated.

## 3. Results

Forty-six subjects were included in the data analysis. In the quiet environment, the total monitoring time was 13.3 hours, including 499 events of apnea. In the noisy environment, the total monitoring time was 13.2 hours, including 492 events of apnea.  Table 1 showed the demographics of the 46 subjects.

The mixed signal *s*(*n*) was created using the 20 sec uncontaminated tracheal sounds and the 20 sec weighted noise signal. The SNRo were -1.35 dB, -4.87 dB, and -7.37 dB, respectively, when Gi was set to 1, 1.5, and 2. After the NLMS AF algorithm was applied on *s*(*n*), the SNRf increased to 0.55 dB, 0.82 dB, and 1.32 dB, respectively, which demonstrated that the filtering algorithm could suppress the ambient noise and improve the quality of tracheal sounds under different noise levels, as is shown in Table 2.

**Figure 5:**
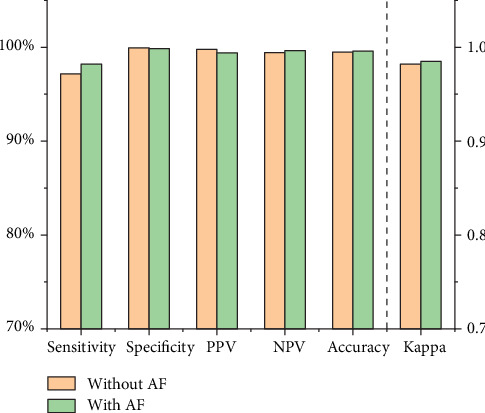
Sensitivity, specificity, PPV, NPV, accuracy, and kappa coefficient of apnea detection algorithm using tracheal sounds without and with AF in the quiet environment.

Table 3 shows that in the quiet environment, the tracheal sounds without and with AF correctly detected apnea 485 times (average length was 21.31 ± 1.85 sec) and 490 times (average length was 21.68 ± 2.13 sec) out of the 499 times (average length was 22.68 ± 1.43 sec) it occurred. Meanwhile, tracheal sounds without and with AF falsely reported apnea 1 and 3 times, respectively, during the period that contained 2488 nonapnea events.  Figure 5 presents the apnea detection performance using tracheal sounds in the quiet environment. The tracheal sounds without AF detected apnea with 97.2% sensitivity, 99.9% specificity, 99.8% PPV, 99.4% NPV, and 99.5% accuracy in the quiet environment. The kappa coefficient was 0.982. The tracheal sounds with AF detected apnea with 98.2% sensitivity, 99.9% specificity, 99.4% PPV, 99.6% NPV, and 99.6% accuracy in the quiet environment. The kappa coefficient was 0.985.

**Figure 6:**
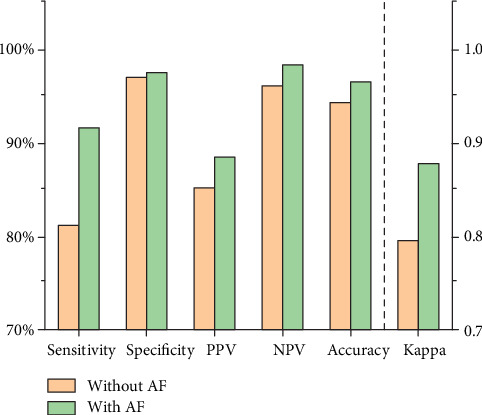
Sensitivity, specificity, PPV, NPV, accuracy, and kappa coefficient of apnea detection algorithm using tracheal sounds without and with AF in the noisy environment.

Table 4 showed that in the noisy environment, the tracheal sounds without and with AF correctly detected apnea 399 times (average length was 21.77 ± 2.61 sec) and 450 times (average length was 21.75 ± 2.27 sec) out of the 492 times (average length was 22.87 ± 1.26 sec) it occurred. Meanwhile, tracheal sounds without and with AF falsely reported apnea 70 and 59 times, respectively, during the period that contained 2300 nonapnea events.  Figure 6 illustrated the apnea detection performance using tracheal sounds in the noisy environment. The tracheal sounds without AF detected apnea with 81.1% sensitivity, 96.9% specificity, 85.1% PPV, 96% NPV, and 94.2% accuracy in the noisy environment. The kappa coefficient was 0.795. The tracheal sounds with AF detected apnea with 91.5% sensitivity, 97.4% specificity, 88.4% PPV, 98.2% NPV, and 96.4% accuracy in the noisy environment. The kappa coefficient was 0.877.

## 4. Discussion

In this study, tracheal sounds collected from the subjects in the quiet and noisy environments were used to detect apnea. The final results showed that in the quiet environment, tracheal sounds without and with AF had similar apnea detection performance, and in the noisy environment, the NLMS AF algorithm significantly improved the performance of apnea detection using tracheal sounds. The validity of the AF algorithm in apnea detection using tracheal sounds was verified.

According to the difference of the AF algorithm optimization criterion, the AF algorithm can be divided into least mean square (LMS), Recursive Least Square (RLS), NLMS, etc. Compared with the LMS algorithm, the NLMS algorithm maintains a higher rate of convergence no matter whether the input signals are correlative or not, and it provides a better trade-off between convergence speed and computational complexity. Meanwhile, the RLS algorithm was used in our pilot study, and it showed similar apnea detection performance in comparison with the NLMS algorithm. However, the real-time processing for tracheal sounds of the RLS algorithm can be limited because of the complicated calculation, the large storage space, and the long program running time. Therefore, the NLMS algorithm was chosen to process tracheal sounds in our study.

When the tracheal sounds were recorded in the noisy environment, the speaker played the TV show using a constant volume. However, the amplitude of noise is uncontrollable in practice. Hence, we introduced Gi to adjust the proportion of the noise in the synthetic signal *s*(*n*) which was created using *x*(*n*) and *B*(*n*). Then, we calculated the improvement of SNR before and after filtering to describe the noise filtering performance of the NLSM AF algorithm when Gi was set to 1, 1.5, and 2, respectively. The results showed that the improvement of SNR became more obvious with the increment of Gi which indicated that the NLMS AF algorithm could perform well in different kinds of noise environments.

In the quiet environment, the unfiltered and filtered tracheal sounds detected apnea with similar sensitivity, specificity, PPV, and NPV. This was because when we applied the NLMS AF algorithm on the tracheal sounds in the quiet environment, the reference input signal was almost zero which resulted in the desired signals (the filtered tracheal sounds) being basically the same with the unfiltered tracheal sounds. In the noisy environment, the apnea detection sensitivity of the filtered tracheal sounds increased from 81.1% to 91.5% compared with the unfiltered tracheal sounds. The proposed filtering algorithm suppressed the noise in tracheal sounds which might be detected as an inspiration or expiration when apnea occurred, and accordingly, the fewer missed events of apnea (from 93 to 42) resulted in the improvement of sensitivity. Meanwhile, the filtered tracheal sounds obtained higher Cohen's kappa coefficient (0.877 versus 0.795) in apnea detection compared with unfiltered tracheal sounds. These results suggested that the proposed filtering algorithm provided a better performance in apnea detection using tracheal sounds.

The volunteers in our study held their breath with their own ways to simulate the events of apnea. The PSG data review showed that some volunteers simulated “central” apnea with an absence of respiratory effort, and the other volunteers simulated “obstructive” apnea using the pattern similar with the Mueller maneuver [23] where participants' attempt at inspiration was made with closed mouth and nose after a forced expiration. This meant that the simulated events of apnea were approximately “obstructive” and “central.” Therefore, we employed our previous tracheal sound apnea detection algorithm. Although it has some limitations in detecting hypopnea and severe airway obstruction and in diagnosing the severity of apnea, the algorithm proved to be reliable and accurate in detecting complete obstructive and central apnea during anesthesia [1, 2].

In these previous studies [1, 2], noise in tracheal sounds was detected as a normal breath when actual apnea appeared. Persistent noise in tracheal sounds raised the log-var threshold and resulted in false apnea alarms. According to the results in this study, although the secondary analysis of these previous studies cannot be conducted because of the lack of a reference signal, we feel that the apnea detection performance in these previous studies would further improve if the proposed AF algorithm was performed. Apnea was defined as a cessation of breath longer than 15 sec in our study. The criterion for apnea is also from our previous studies [1, 2]. Although the sleep-related breathing disorder studies usually used 10 sec as the threshold, the conclusion of our study would not change, especially for the validity of the AF algorithm.

Although many approaches have been investigated in screening OSA during wakefulness using tracheal sounds [3–6], none of these studies developed its algorithm in the noisy environment. In these studies, a considerable proportion (1/3 to 1/2) of tracheal sound data from the subjects was excluded from analysis because of noise contamination, which narrowed the application of these algorithms. Hence, we feel that these studies would achieve more application scenarios and less application restrictions if the proposed AF algorithm was introduced.

In our experiment, the quiet environment was just defined by the researchers subjectively and may contain some inherent noise actually. We did not provide a quantitative description of a quiet environment. Therefore, SNRo and SNRf may not be absolutely accurate. However, we feel the conclusion from SNR comparison will not change because the NLMS AF algorithm could suppress the noise in tracheal sounds and improve the tracheal sounds quality under different noise levels. A quantitative description of noisy environment was also not provided. The noise was generated using a speaker which could lead to missed events of apnea detected by tracheal sounds. In practice, the type and level of noise might be different from our experiment. Hence, validating and optimizing the AF algorithm in real-life medical settings may be necessary to improve the quality of the recorded tracheal sounds and result in better performance of apnea detection.

## 5. Conclusions

The feasibility and validity of the NLMS AF algorithm which was applied on tracheal sounds have been verified under different noise levels. This filtering algorithm can effectively improve the quality of the recorded tracheal sounds in the noisy environment and result in better performance of apnea detection. Thus, the AF technology should be integrated into the application of real-time respiratory monitoring using tracheal sounds.

## Figures and Tables

**Table 1:** Demographic data of 46 subjects.

	Mean ± SD	Min	Max
Male/female	24/22		
Smoking/no smoking	2/44		
Age (year)	22.3 ± 2.3	18	35
Height (cm)	168.9 ± 8.7	153	186
Weight (kg)	61.6 ± 13.1	42	105
BMI (kg/m^2^)	21.4 ± 2.9	17.1	31.4
Neck circumference	36.2 ± 3.4	31	47

**Table 2:** The calculated SNR before and after filtering.

Gi	SNRo (dB)	SNRf (dB)	The improvement of SNR
1	-1.35	0.55	1.9
1.5	-4.87	0.86	5.73
2	-7.37	1.32	8.69

**Table 3:** The number of times apnea was detected by tracheal sounds and the PSG deriving signal in the quiet environment.

	PSG deriving signal	
	Apnea	Normal
Tracheal sounds without AF		
Apnea	485 times (TP)	1 time (FP)
Normal	14 times (FN)	2487 times (TN)
Tracheal sounds with AF		
Apnea	490 times (TP)	3 times (FP)
Normal	9 times (FN)	2485 times (TN)

TP = true positive; FP = false positive; FN = false negative; TN = true negative.

**Table 4:** The number of times apnea was detected by tracheal sounds and the PSG deriving signal in the noisy environment.

	PSG deriving signal	
	Apnea	Normal
Tracheal sounds without AF		
Apnea	399 times (TP)	70 times (FP)
Normal	93 times (FN)	2230 times (TN)
Tracheal sounds with AF		
Apnea	450 times (TP)	59 times (FP)
Normal	42 times (FN)	2241 times (TN)

TP = true positive; FP = false positive; FN = false negative; TN = true negative.

## Data Availability

The (healthy subjects) data used to support the findings of this study are available from the corresponding author upon request.
